# Peripheral NF-κB dysregulation in people with schizophrenia drives inflammation: putative anti-inflammatory functions of NF-κB kinases

**DOI:** 10.1038/s41398-021-01764-2

**Published:** 2022-01-13

**Authors:** Caitlin E. Murphy, Adam K. Walker, Maryanne O’Donnell, Cherrie Galletly, Andrew R. Lloyd, Dennis Liu, Cynthia Shannon Weickert, Thomas W. Weickert

**Affiliations:** 1 Neuroscience Research Australia, Sydney, New South Wales Australia; 2 School of Psychiatry, University of New South Wales, Sydney, Australia; 3 Drug Discovery Biology Theme, Monash University, Parkville, Australia; 4 Discipline of Psychiatry, School of Medicine, University of Adelaide, Adelaide, South Australia Australia; 5 Northern Adelaide Local Health Network, Adelaide, South Australia Australia; 6 Ramsay Health Care (SA) Mental Health, Adelaide, South Australia Australia; 7 Kirby Institute, University of New South Wales, Sydney, Australia; 8 Department of Neuroscience & Physiology, Upstate Medical University, Syracuse, New York, NY USA

**Keywords:** Neuroscience, Biomarkers

## Abstract

Elevations in plasma levels of pro-inflammatory cytokines and C-reactive protein (CRP) in patient blood have been associated with impairments in cognitive abilities and more severe psychiatric symptoms in people with schizophrenia. The transcription factor nuclear factor kappa B (NF-κB) regulates the gene expression of pro-inflammatory factors whose protein products trigger CRP release. NF-κB activation pathway mRNAs are increased in the brain in schizophrenia and are strongly related to neuroinflammation. Thus, it is likely that this central immune regulator is also dysregulated in the blood and associated with cytokine and CRP levels. We measured levels of six pro-inflammatory cytokine mRNAs and 18 mRNAs encoding NF-κB pathway members in peripheral blood leukocytes from 87 people with schizophrenia and 83 healthy control subjects. We then assessed the relationships between the alterations in NF-κB pathway genes, pro-inflammatory cytokine and CRP levels, psychiatric symptoms and cognition in people with schizophrenia. IL-1β and IFN-γ mRNAs were increased in patients compared to controls (both *p* < 0.001), while IL-6, IL-8, IL-18, and TNF-α mRNAs did not differ. Recursive two-step cluster analysis revealed that high levels of IL-1β mRNA and high levels of plasma CRP defined ‘high inflammation’ individuals in our cohort, and a higher proportion of people with schizophrenia were identified as displaying ‘high inflammation’ compared to controls using this method (*p* = 0.03). Overall, leukocyte expression of the NF-κB-activating receptors, TLR4 and TNFR2, and the NF-κB subunit, RelB, was increased in people with schizophrenia compared to healthy control subjects (all *p* < 0.01), while NF-κB-inducing kinase mRNAs IKKβ and NIK were downregulated in patients (all *p* < 0.05). We found that elevations in TLR4 and RelB appear more related to inflammatory status than to a diagnosis of schizophrenia, but changes in TNFR2 occur in both the high and low inflammation patients (but were exaggerated in high inflammation patients). Further, decreased leukocyte expression of IKKβ and NIK mRNAs was unique to high inflammation patients, which may represent schizophrenia-specific dysregulation of NF-κB that gives rise to peripheral inflammation in a subset of patients.

## Introduction

**Fig. 1:**
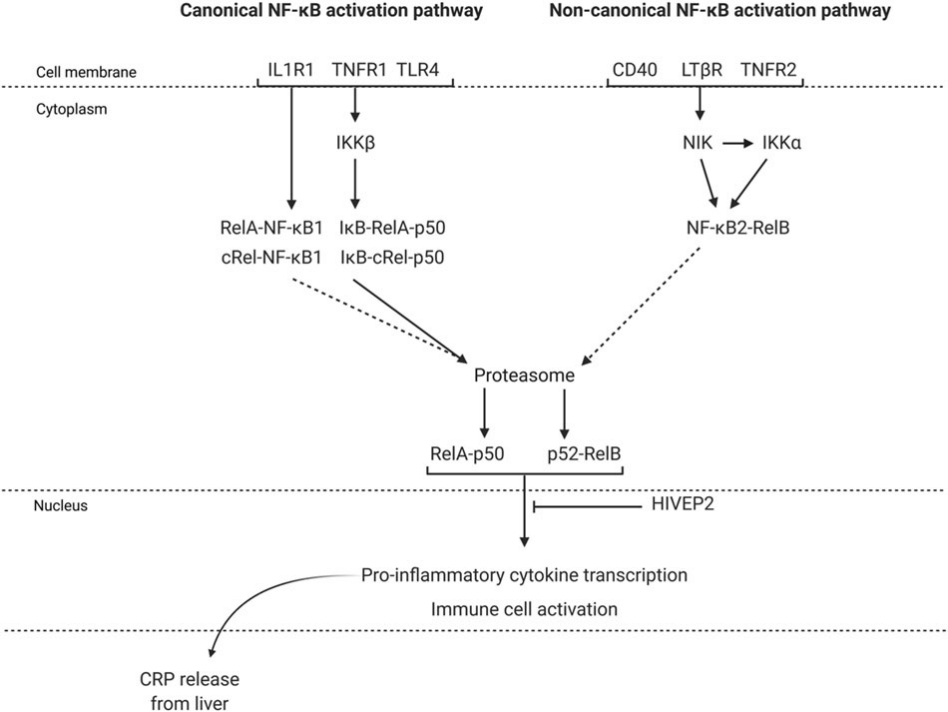
*NF-κB activation*. In canonical NF-κB activation, immunoreceptors such as interleukin-1 receptor type 1 (IL1R1), tumor necrosis factor receptor superfamily member 1 A (TNFR1) and toll-like receptor 4 (TLR4) activate inhibitor of NF-κB kinase subunit beta (IKKβ). IKKβ then tags inhibitor of NF-κB (IκB) for proteasomal degradation, freeing the transcriptionally active NF-κB dimer made of p50 and RelA/cRel proteins. Activation of canonical NF-κB receptors also enhances the partial processing of p50 precursor NF-κB1 into p50. As a result of NF-κB1 processing or IκB degradation, the p50-RelA/cRel dimer moves into the nucleus where it initiates pro-inflammatory gene transcription. In noncanonical NF-κB activation, ligand-binding of immunoreceptors, such as cluster of differentiation 40 (CD40), lymphotoxin receptor beta (LTβR), and TNFR superfamily member 1B (TNFR2) leads to stabilization of NF-κB inducing kinase (NIK) and activation of IKKα. These kinases then tag RelB-bound NF-κB2 for partial proteasomal processing into p52. The p52-RelB dimer moves into the nucleus where it initiates pro-inflammatory gene transcription. Though the DNA-binding affinities of NF-κB dimers are largely overlapping [26], canonical NF-κB activation is rapid and typically transient, whereas activation of the non-canonical NF-κB pathway is characteristically slower and more persistent [27]. HIVEP2 is a nuclear protein that inhibits the binding of NF-κB dimers to DNA. High levels of proinflammatory cytokine proteins in the blood trigger CRP release from the liver into the bloodstream [28]. Dotted lines to the proteasome indicate partial processing (NF-κB1 and NF-κB2).

The role of inflammation in the pathophysiology of schizophrenia is well-supported by consistent findings of elevated inflammatory markers in both the postmortem brain of individuals who suffered from schizophrenia and in the blood of living patients [1–7]. Further, several whole-genome and RNA-sequencing studies have found changes in the immune system of patients in the brain and blood [8–10], though few studies have identified specific aspects of immunoregulatory pathways that may be causing such aberrations. While it is not currently known what drives this presumed noninfective inflammation in schizophrenia, overactivity of the immune regulator nuclear factor kappa B (NF-κB) has recently been proposed to prime patients for exaggerated and prolonged immune responses in the brain [3, 11–14]. Consistent with this, we and others have found altered expression of NF-κB pathway mRNAs in the post-mortem cerebral cortex of people with schizophrenia [12, 14], and we showed that individuals with increased levels of NF-κB-inducing mRNAs also have increased levels of pro-inflammatory cytokine transcripts in the brain (for a detailed explanation of NF-κB-signaling pathways, see Fig. 1). However, the inflammation-associated increase in NF-κB pathway transcripts appears blunted in patients relative to nonschizophrenic controls, specifically in regard to the pattern recognition receptor toll-like receptor 4 (TLR4) which is enriched in brain resident macrophages (microglia) and crucial in the activation of the pro-inflammatory microglial phenotype. Conversely, peripheral monocytes/macrophages appear to be overactive in some people with schizophrenia, evidenced by elevated blood levels of proinflammatory, macrophage-derived cytokines [15–18], and reported increases in TLR4 mRNA in patient leukocytes [19, 20]. When patient leukocytes are isolated, cultured and exposed to lipopolysaccharide (LPS; which activates NF-κB in monocytes/macrophages via TLR4), they show exaggerated inflammatory responses compared to cells from healthy controls [21]. Taken together, existing literature supports that there may be distinct molecular inflammatory changes in NF-κB in the brain versus blood, and that there may be an increased propensity for monocyte/macrophage-mediated inflammation in the periphery in schizophrenia.

The subset of patients that show evidence of immune activation (as assayed in the blood) suffer from more severe cognitive symptoms than those without active inflammation [18, 22–25], highlighting the clinical relevance of peripheral immune activation to schizophrenia symptomatology. Further, plasma levels of the acute phase protein C-reactive protein (CRP) are inversely correlated with prefrontal cortex (PFC) thickness in schizophrenia [25], which might suggest that the PFC is a brain region particularly vulnerable to peripherally derived inflammatory insult. Previous attempts to identify ‘inflamed’ patients have used blood levels of CRP [22–25] or blood levels of pro-inflammatory cytokine transcripts [5, 18], both of which are directly (cytokine transcription) or indirectly (CRP is released from the liver into the blood) triggered by NF-κB activation (Fig. 1).

We hypothesized that peripheral inflammation in this ‘high inflammation’ patient subgroup may be linked to schizophrenia-specific overexpression of transcripts that activate NF-κB, including TLR4, in leukocytes. Stratification using plasma CRP or levels of pro-inflammatory cytokine transcripts in isolation have identified patient subgroups with more severe symptoms, poorer cognition and worse neuropathology [18, 22–25], but it is not known whether patients with elevated plasma CRP levels overlap with patients who have increased levels of cytokine transcripts or which specific cytokine upregulations may be most associated with plasma CRP in schizophrenia. Thus, we determined whether combining CRP and inflammatory transcripts together would be a viable way to stratify people into high and low inflammatory groups. Here we ask, for the first time, if the transcriptional levels of any key member of the NF-κB pathway of living patients with schizophrenia differs from healthy control subjects in the cytokine-producing leukocytes. We then assessed the extent to which alterations in NF-κB pathway member mRNAs in the blood: 1) occur in all patients or are unique to patients with inflammation; and 2) are schizophrenia-specific or also occur in non-schizophrenic controls with active inflammation (and thus reflect high inflammatory status regardless of diagnosis). Finally, we examined the relationships between altered levels of NF-κB pathway mRNAs, downstream markers of inflammation, psychiatric symptoms and impairment in PFC-dependent cognitive function in patients.

## Methods and materials

### Participants

Ninety-seven people diagnosed with schizophrenia or schizoaffective disorder were recruited from sites in Sydney and Adelaide, Australia, via clinician, self or family referral. All patients were community-dwelling and had been receiving antipsychotic medication for at least 1 year prior to entering the study. A diagnosis of schizophrenia or schizoaffective disorder was determined using the Structured Clinical Interview for Diagnostic and Statistical Manual IV-TR Axis I Disorders (SCID) [26] by a clinician trained in the administration of the SCID and was confirmed independently by another clinician. Patients with a concurrent Axis I psychiatric diagnosis, a history of substance abuse or dependence (within the past 5 years), head injuries with loss of consciousness, seizures, central nervous system infection, untreated diabetes, or hypertension, or mental retardation were excluded. Women were excluded if they were pregnant or were receiving hormone therapy and refused alternate forms of birth control. Eighty-seven healthy control subjects were also recruited through advertisements at the University of New South Wales and in the local communities in Sydney and Adelaide, Australia. Exclusion criteria consisted of a personal history of or a first-degree relative with a DSM-IV Axis I psychiatric diagnosis, history of substance abuse or dependence (within the past 5 years), head injuries with loss of consciousness, seizures, central nervous system infection, untreated diabetes or hypertension or mental retardation. We screened our patients and controls for other medical conditions and none of the patients or controls entering the study reported any history of inflammatory diseases. A medical doctor also collected a history of any medical conditions and performed a physical medical exam at the time of the study. No patients or controls showed any indication of inflammatory processes (e.g. fever, sore throat, lung congestion/difficulty breathing). People with schizophrenia did not differ from control participants in male:female ratio (Table 1), but were on average 6 years older than control participants (*p* = 0.001). Data regarding smoking status was only available for 8 controls and 68 patients, however based on this subset of participants, people with schizophrenia were more likely to be current smokers (Table 1). While the primary purpose of recruitment was for enrollment in a double-blind, placebo-controlled, cross-over trial of adjunctive raloxifene [27], the current study used available leukocyte RNA (83 controls and 87 people with schizophrenia) and plasma (69 controls and 76 people with schizophrenia) extracted from blood samples collected at baseline (prior to the commencement of the trial). About one-third of our patient cohort (36%) were receiving clozapine at the time of the study. The next most commonly used antipsychotics were risperidone (15%) and olanzapine (13%). Antipsychotic medications were converted to chlorpromazine (CPZ) equivalent doses per day [28].

**Table 1:** Demographic and cognitive variables in control and patient participants.

	Control (*N* = 83)	Schizophrenia (*N* = 87)		
			U/F/χ² (df)	*p* value
Age	29.0 (5.0)	35.0 (6.0)	*U* = 2514.50	**0.001**
Sex (m/f)	42/41	54/33	*χ*²(1) = 2.72	0.13
Smoking status (yes/no)	*n* = 8 1/7	*n* = 68 36/32	*χ*²(1) = 4.69	**0.03**
RIN	*n* = 79 7.78 (1.16)	*n* = 84 7.54 (1.09)	*U* = 2845.50	0.12
Working memory	0.02 (1.71)	−1.83 (1.63)	*F*(1,167) = 42.11	< **0.001**
Language	−0.06 (1.69)	−1.18 (1.73)	*F*(1,167) = 14.46	< **0.001**
PANSS positive	–	15.3 (4.6)	–	–
PANSS negative	–	14.5 (6.1)	–	–
PANSS general	–	30.9 (8.8)	–	–
PANSS total		60.7 (16.5)	–	–
CPZ equivalent (mg)/day	–	550.37 (462.51)	–	–
Duration of illness (years)	–	13.00 (7.5)	–	–

*RIN* RNA integrity number, *PANSS* Positive and Negative Syndrome Scale, *CPZ* chlorpromazine.

Data presented as mean (standard deviation) for RIN, working memory and language (combined *z*-scores from cognitive tests). Data presented as median (median absolute deviation) for age. Bold values indicate significance at *p* ≤ 0.01. “U” denotes results of Mann-Whitney test, “*χ*^2^” denotes results of Chi-square test, “*F*” denotes results of univariate ANCOVA.

### Cognitive testing and symptom assessment

As part of a battery of baseline psychometric assessments [27], all participants were administered cognitive subtests that measured PFC-dependent working memory (Letter-Number Sequencing [LNS] and Arithmetic from the Wechsler Adult Intelligence Scale-Third Edition [WAIS-III]) [29] and tests of language abilities (letter fluency from the Controlled Oral Word Association Test [COWAT] [30] and WAIS-III Similarities). Assessments were made by a psychologist or psychometrician trained in administration and scoring. The 2 working memory test scores were converted to *z*-scores and summed, and the 2 language test scores were converted to *z*-scores and summed, to provide separate overall scores for working memory and language respectively. Symptom severity was assessed in patients at baseline using the Positive and Negative Syndrome Scale (PANSS) [31].

### Blood collection and processing

Peripheral venous blood was collected from all participants in the morning between 9 and 11 am. For RNA extraction, blood was collected in 9 mL acid citrate dextrose (ACD-B) tubes (BD Biosciences, North Ryde, New South Wales, Australia). Total RNA was extracted using the Trizol method (Invitrogen, Carlsbad, CA, USA) and RNA quality and concentration were assessed on the Agilent Technologies 2100 Bioanalyzer and Nanodrop ND-1000 spectrophotometer. Four control and 3 patient samples had low RIN values < 4 and were excluded from RNA analysis. RIN values were not significantly different between diagnostic groups (Table 1). cDNA was synthesized from 1 μg total RNA per case with the Invitrogen Superscript IV kit (Invitrogen, Carlsbad, CA, USA) and random hexamers following manufacturer instructions (Life Technologies). For plasma assays, blood was collected in 9 mL ethylenediaminetetraacetic acid (EDTA) tubes (Vacuette Vacutainer, Greiner Bio-One, Kremsmünster, Austria). Plasma was collected from EDTA tubes via centrifugation for 15 min at 2000 x *g* and aliquoted into protein low-binding tubes (Eppendorf, Hamburg, Germany) and stored at −80°C until the day of the assay.

### Quantitative real-time PCR

The mRNA expression of 6 cytokine and 18 NF-κB pathway genes was measured by reverse transcriptase–qPCR via the Fluidigm® BioMark™ HD system (South San Francisco, CA, USA) at the Ramaciotti Centre for Genomics (Kensington, NSW, Australia), including: interleukin (IL)-6, IL-1β, IL-8, IL-18, tumor necrosis factor alpha (TNFα), interferon-gamma (IFN-γ), IL-1 receptor type 1 (IL1R1), TNF receptor superfamily member 1 A (TNFR1), TLR4, cluster of differentiation 40 (CD40), lymphotoxin beta receptor (LTβR), TNFR2, inhibitor of NF-κB kinase subunit alpha (IKKα), IKK beta (IKKβ), NF-κB-inducing kinase (NIK), inhibitor of NF-κB alpha (IκBα), IκB beta (IκBβ), IκB epsilon (IκBε), Human Immunodeficiency Virus Enhancer Protein 2 (HIVEP2), RelA, RelB, cRel, NF-κB subunit 1 (NF-κB1), and NF-κB2. The following predesigned Taqman Gene Expression Assays were used: IL-6 (Hs00174131_m1), IL-1β (Hs01555410_m1), IL-8 (Hs00174103_m1), IL-18 (Hs01038788_m1), TNFα (Hs00174128_m1), IFN-γ (Hs00989291_m1), IL1R1 (Hs00991010_m1), TNFR1 (Hs01042313_m1), TLR4 (Hs00152939_m1), CD40 (Hs00374176_m1), LTβR (Hs01101194_m1), TNFR2 (Hs00961750_m1), IKKα (Hs00989497_m1), IKKβ (Hs01559460_m1), NIK (Hs01089753_m1), IκBα (Hs00153283_m1), IκBβ (Hs00182115_m1), IκBε (Hs00234431_m1), HIVEP2 (Hs00198801_m1), RelA (Hs01042014_m1), RelB (Hs00232399_m1), cRel (Hs00968440_m1), NF-κB1 (Hs00765730_m1), and NF-κB2 (Hs00174517_m1). Controls included no reverse transcriptase and no template to rule out genomic DNA contamination and reagent contamination, respectively. Normalized relative quantities (2-ΔΔCT) of each mRNA were calculated using the geometric mean of four housekeeper genes (ACTB, GAPDH, TBP, UBC) that did not differ between diagnostic groups (all *p* > 0.32) (ACTB, Hs99999903_m1; GAPDH, Hs99999905_m1; TBP, Hs00427620_m1; UBC, Hs00824723_m1).

### Plasma C-reactive protein (CRP) assay

CRP was measured in plasma from 69 controls and 76 people with schizophrenia using a high-sensitivity enzyme-linked immunosorbent assay (ELISA) according to the manufacturer’s instructions (IBL-international, Hamburg, Germany). Ten microliters of plasma were diluted serially up to 1:1000 and each assay was run back-to-back with samples in duplicate by the same investigator, who was blind to the diagnosis. A five-point standard curve was generated using 0, 0.4, 1, 5, and 10 mg/L calibrators that were prepared by the manufacturer. The average coefficient of variance across all samples was 3.23%. The sample reads ranged from 0.03 mg/L to 23.93 mg/L, with the minimum value replacement of 0.03 mg/L used for 12 data points that were below the minimal detectable value. As population studies have shown that CRP measured in the blood of healthy subjects is typically skewed to the right and non-normally distributed [32], the 95th percentile value was used as the upper limit cut-off [33] for diagnostic comparisons. Seven values (1 control, 6 patients) above this cut-off (16.23 mg/L) were excluded from diagnostic analyses.

### Statistical analyses

Independent samples *t* tests, Mann-Whitney *U* tests, univariate ANCOVAs or Chi-square tests were used to assess differences in age, RNA integrity number (RIN), male:female ratio between controls and patients (Table 1). ANCOVAs with cognitive domain score as the dependent variable, diagnosis as the grouping variable and age as a covariate were used to compare working memory and language abilities between controls and patients (Table 1).

Cytokine and NF-κB pathway member mRNAs that were not normally distributed were log-transformed to achieve normality. Normalized relative mRNA quantities >2 standard deviations from the diagnostic group mean were considered outliers and excluded from analyses (on average two controls and four patients/transcript). To assess the effects of age and RIN on our mRNAs of interest, correlations were performed between each mRNA and RIN, and each mRNA and age. To assess the effects of body mass index (BMI) on our inflammatory variables of interest, correlations with performed between each variable and BMI in patients for whom we had available BMI data (*n* = 68). BMI data was only available for eight control participants and so was not analysed in controls. Independent samples t-tests or one-way ANCOVAs were used to assess differences in peripheral leukocyte expression of IL-6, IL-1β, IL-8, TNFα, and IFN-γ mRNAs and each NF-κB-related mRNA between controls and patients, with mRNA as the dependent variable, diagnosis as the grouping variable and RIN and/or age as covariate (if correlated with the mRNA of interest in either group; Supplementary Table 1). For schizophrenia patients, correlations were performed with mRNA levels and mean daily chlorpromazine equivalent dose to determine any relationship between expression of each transcript and antipsychotic exposure. In order to test the hypothesis that NF-κB regulation may have unique changes relative to inflammation and/or an altered relationship to inflammation in only a subset of patients, we performed recursive two-step clustering with the entire cohort using plasma CRP (including those above 16.23 mg/L) and peripheral leukocyte levels of IL-6, IL-1β, IL-8, IL-18, TNFα, and IFN-γ mRNAs as potential clustering factors. No decision about the number of clusters to be generated was made a priori, and the overall model quality was required to be >0.5, with predictors removed if they did not contribute >0.5 to the model on a scale of 0–1.0. We then compared NF-κB pathway mRNAs between high and low inflammation biotypes within each diagnosis, and tested for diagnosis and inflammation interactions with two-way ANCOVAs, with diagnosis and inflammation status (high/low) as the grouping variables. We used planned post-hoc comparisons between subgroups to determine whether changes in these mRNAs were unique to schizophrenia patients with elevated inflammatory markers. To assess the relationship between NF-κB dysregulation and downstream inflammation in schizophrenia, we ran correlations between NF-κB pathway mRNAs that were uniquely altered in high inflammation schizophrenia patients and the markers identified by the model as defining a heightened inflammatory state. Finally, we tested whether high and low inflammation patient subgroups differed in the severity of symptoms using two-tailed *t* tests for PANSS scores and cognitive domain scores. An alpha value of 0.05 was used for all statistical tests, and for diagnostic comparisons of our primary 25 immune variables of interest (CRP, six cytokine mRNAs and 18 NF-κB pathway mRNAs), the Holm-Bonferroni sequential correction was applied to control for multiple comparisons [34].

## Results

### Cohort demographics

People with chronic schizophrenia displayed mild-to-moderate symptom severity on the PANSS. Patients had significantly poorer language and working memory composite scores (Table 1) and higher plasma CRP than control participants (Cohen’s *d* = 0.58, *p* < 0.001, adjusted *p* < 0.03; Table 2), as reported in previous studies [25, 27]. People with schizophrenia also had approximately 25% higher leukocyte IL-1β mRNA (Cohen’s *d* = 0.63) and 42% higher IFN-γ (Cohen’s *d* = 0.65) mRNA than controls (both p < 0.001, both adjusted *p* < 0.02; Table 2). No diagnostic difference was detected in IL-6, IL-8, IL-18, or TNFα mRNAs (all *p* > 0.22, Table 2). In patients, IL-1β mRNA was weakly, inversely correlated with antipsychotic dose (Spearman’s *ρ* = −0.24, *p* = 0.03; Supplementary Figure 1), whereas CRP and IFN-γ, IL-6, IL-8, IL-18, and TNFα mRNAs did not correlate with antipsychotic dose (chlorpromazine equivalent dose; all Spearman’s *ρ* < |0.21 | , all *p* > 0.08). In patients, CRP was weakly positively correlated with BMI, and IFN-γ mRNA was weakly negatively correlated with BMI (Supplementary Table 2). IL-1β, IL-6, IL-8, IL-18, and TNFα mRNAs were not correlated with BMI in patients.

**Table 2:** Comparison of plasma CRP and proinflammatory cytokine mRNA levels between diagnostic groups.

	Control (*N* = 83)	Schizophrenia (*N* = 87)		
			t/U/F (df)	*p* value
Plasma CRP (mg/L)	*n* = 68 0.55 (0.52)	*n* = 70 2.26 (1.60)	*U* = 1340.00	<**0.001**
IL-6 mRNA	0.96 (0.45)	1.07 (0.52)	*t*(148) = −1.23	0.22
IL-1β mRNA	0.83 (0.25)	1.04 (0.35)	*t*(149) = −4.19	**<0.001**
IL-8 mRNA	1.22 (0.57)	1.17 (0.54)	*t*(147) = 0.41	0.68
IL-18 mRNA	1.21 (0.29)	1.27 (0.33)	*F*(1,149) = 0.33	0.57
TNF-α mRNA	0.95 (0.21)	0.95 (0.29)	*F*(1,147) = 0.76	0.39
IFN-γ mRNA	0.89 (0.43)	1.26 (0.67)	*F*(1,146) = 14.66	<**0.001**

*CRP* C-reactive protein, *IL* interleukin, *TNF* tumor necrosis factor, *IFN* interferon.

Data presented as mean (standard deviation) for IL-6, IL-1β, IL-8, IL-18, TNFα, and IFN-γ mRNAs (2−^∆∆CT^). Data presented as median (median absolute deviation) for plasma CRP. Bold values indicate significance at *p* < 0.001. *P* values for CRP, IL-1β mRNA, and IFN-γ mRNA remain significant after Holm-Bonferroni correction for multiple comparisons (all adjusted *p* ≤ 0.03).

### People with schizophrenia have increased levels of NF-κB-activating receptor mRNAs in peripheral leukocytes

**Fig. 2:**
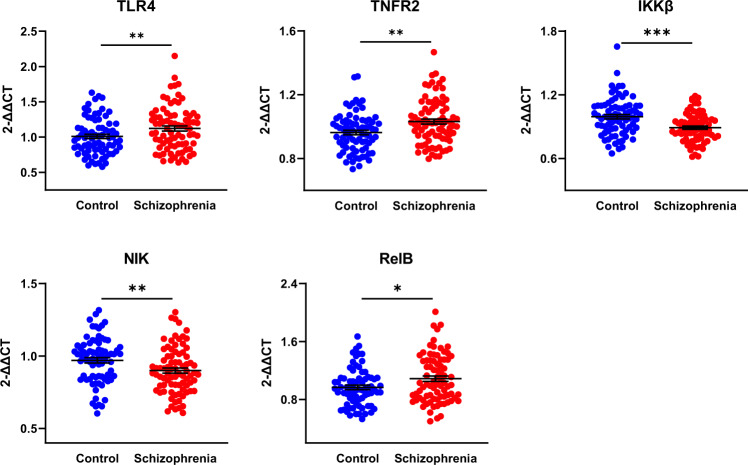
Peripheral blood leukocyte gene expression of NF-κB pathway transcripts in healthy controls and people with schizophrenia. Lines and whiskers in graphs represent mean + standard error. ***unadjusted *p* < 0.001, **unadjusted *p* < 0.01, *unadjusted *p* < 0.05. The diagnostic difference in IKKβ mRNA remained significant after multiple comparisons correction (adjusted *p* < 0.02).

We found increased mRNA levels of two receptors that activate NF-κB, TLR4 ( + 11%; *F*(1,151) = 8.15, Cohen’s *d* = 0.40, *p* = 0.005, adjusted *p* = 0.10) and TNFR2 ( + 7%; *F*(1,154) = 8.21, Cohen’s *d* = 0.47, *p* = 0.005, adjusted *p* = 0.10) in people with schizophrenia compared to controls. No significant diagnostic difference was detected for IL1R1, TNFR1, CD40, or LTβR mRNAs (all *F* < 1.01, all *p* > 0.32) (Fig. 2). No receptor mRNA level was associated with antipsychotic dose (all *r* < |0.22 | , *p* > 0.05) or BMI (Supplementary Table 2) in patients.

### People with schizophrenia have decreased levels of NF-κB kinase mRNAs in peripheral leukocytes

In contrast to NF-κB pathway receptors, we found that leukocytes from people with schizophrenia had, on average, 10% less IKKβ mRNA than leukocytes from controls (*F*(1,151) = 15.34, Cohen’s *d* = 0.65, *p* < 0.001, adjusted *p* < 0.02). We found a similar effect for NIK mRNA in patient leukocytes (-8%; *F*(1,157) = 8.68, Cohen’s *d* = 0.44, *p* = 0.004, adjusted *p* = 0.08) (Fig. 2). No kinase mRNA was associated with antipsychotic dose (all *r* < |0.14 | , *p* > 0.21) or BMI (Supplementary Table 2) in patients.

### Peripheral blood leukocyte levels of NF-κB inhibitor mRNAs are unchanged in people with schizophrenia

We next analysed gene expression of 3 cytoplasmic inhibitors of canonical NF-κB: IκBα, IκBβ and IκBε – and a nuclear repressor of NF-κB DNA-binding, HIVEP2. All 3 IκB transcripts were unchanged between diagnoses (all *F* < 2.09, all *p* > 0.15) as was HIVEP2 transcript (*F*(1,152) = 2.09, *p* = 0.15). No inhibitor mRNA was associated with antipsychotic dose in patients (all *r* < |0.21 | , all *p* > 0.07). IκBε was weakly, positively correlated with BMI in patients (Supplementary Table 2).

### Peripheral blood leukocyte levels of NF-κB subunits in people with schizophrenia

We found that levels of RelB were increased in patients compared to controls (+12%; *F*(1,153) = 5.47, Cohen’s *d* = 0.41, *p* = 0.02, adjusted *p* = 0.40; Fig. 2). Levels of RelA, cRel, NF-κB1, and NF-κB2 mRNA did not differ between diagnostic groups (all *F* < 2.22, all *p* > 0.14). RelA mRNA level was weakly, inversely correlated with antipsychotic dose (*ρ* = −0.28, *p* = 0.01) and RelB mRNA was weakly, positively correlated with BMI in patients (Supplementary Table 2). No other subunit-encoding mRNA was associated with antipsychotic dose (all *r* < |0.14 | , all *p* > 0.21).

### Peripheral blood leukocyte IL-1β mRNA and plasma CRP discriminate between high and low inflammation controls and people with schizophrenia

**Fig. 3:**
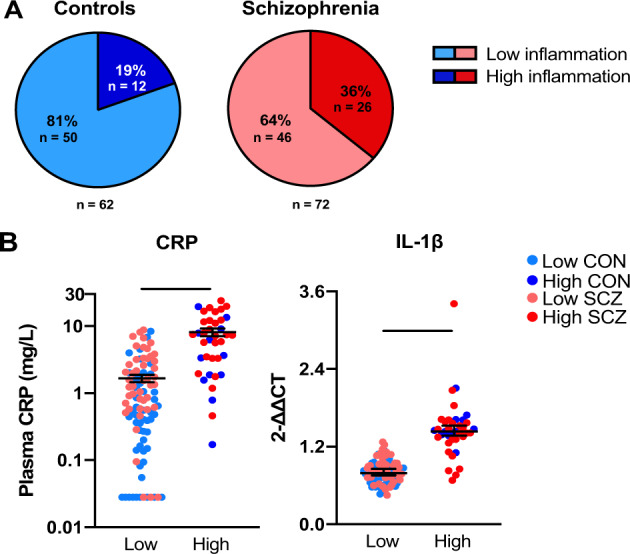
High and low inflammation subgroups. **A** Proportions of control and patient samples that were classified via recursive two-step cluster analysis as having high and low inflammation, based on plasma levels of CRP and peripheral blood leukocyte expression of IL-1β mRNA. **B** Plasma CRP and IL-1β mRNA in high and low inflammation biotypes (median + 95% confidence interval). ***p < 0.001.

Recursive two-step cluster analysis generated 2 inflammatory subgroups, high (*n* = 38) and low (*n* = 96), that were best defined by two factors, IL-1β mRNA and plasma CRP, with an average silhouette value of 0.6. No other cytokine mRNA was necessary to aid in the discrimination (did not significantly contribute >0.5 to the model on a scale of 0–1.0). We subsequently confirmed that leukocyte levels of IL-1β mRNA and plasma CRP were positively correlated (Spearman’s *ρ* = 0.37, *p* < 0.001). The median plasma CRP in the high inflammation group (5.71 mg/mL) was 6.8 times that of the low inflammation group (0.84 mg/mL), and this fits well with the clinical definition of a “noninflamed” individual from the perspective of CRP alone (<1.0 mg/mL) (Fig. 3). The median relative IL-1β mRNA (2−^∆∆CT^) in the high inflammation group (1.45) was almost double (1.8 times) that of the low inflammation group (0.79) (Fig. 3). The proportion of patients classified as having high inflammation (36%) using this model was significantly higher than that of controls (19%) (*X*^2^(1) = 4.60, *p* = 0.03). Smokers were no more likely than non-smokers to be classified as having high inflammation (*X*^2^(1) = 0.72, *p* = 0.40). Comparisons of demographic and clinical variables between high and low inflammation patients can be found in Supplementary Table 3.

### Altered leukocyte expression of IKKβ and NIK mRNAs are unique to people with schizophrenia who also have elevated markers of inflammation

For 14 of the 18 NF-κB transcripts measured, there was a main effect of inflammatory biotype such that high inflammation individuals had higher levels of TNFR1, TLR4, LTβR, TNFR2, IκBα, IκBβ, IκBε, RelB, NF-κB1, and NF-κB2 mRNAs (main effect of inflammation: all *F* > 4.07, all *p* < 0.05) and lower levels of IKKβ, NIK, RelA and cRel mRNAs (main effect of inflammation: all *F* > 3.83, all *p* ≤ 0.05) than low inflammation individuals.

**Fig. 4:**
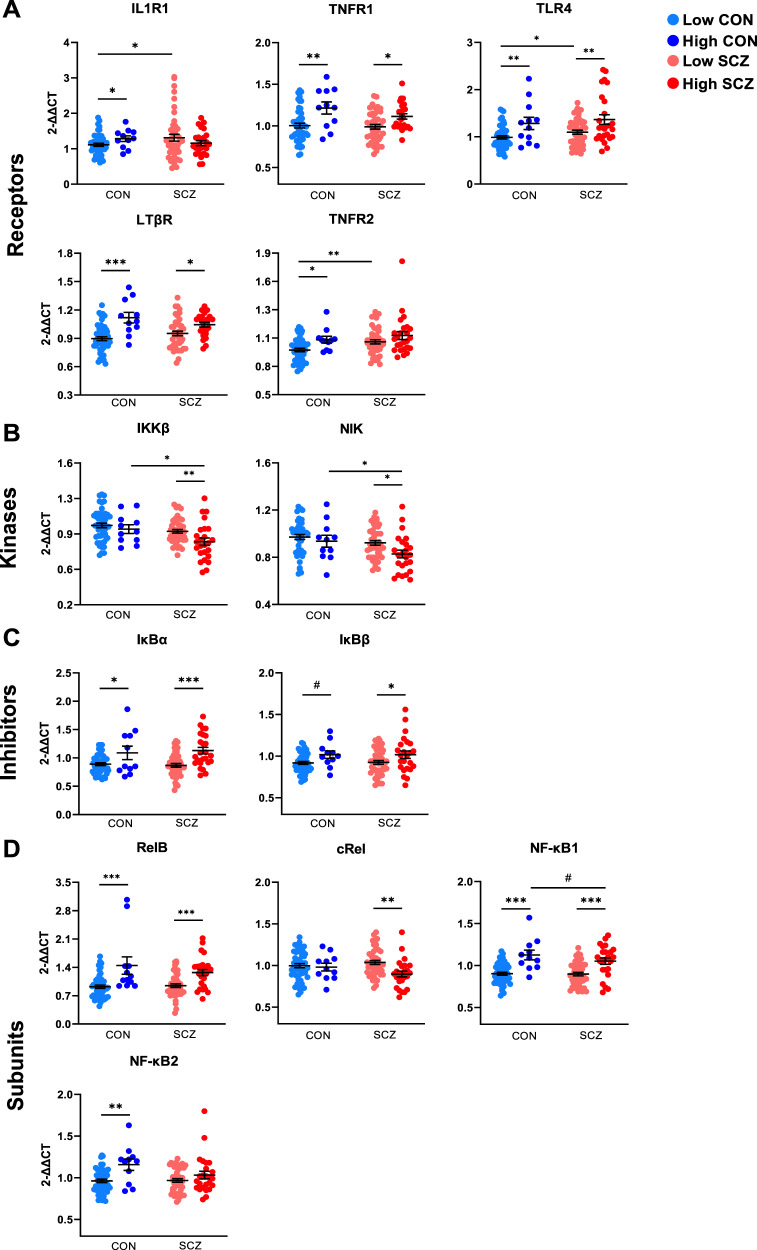
Peripheral blood leukocyte gene expression of the NF-κB pathway in healthy controls and people with schizophrenia stratified by inflammatory biotype. **A** NF-κB-activating receptor mRNAs, **B** NF-κB-inducing kinase mRNAs, **C** NF-κB inhibitor mRNAs, **D** NF-κB subunit-encoding mRNAs. Lines and whiskers in graphs represent mean + standard error. ***p < 0.001, **p < 0.01, *p < 0.05, #*p* = 0.06.

Even after accounting for inflammatory status, people with schizophrenia differed significantly from controls in their expression of TNFR2, IKKβ and NIK mRNAs (main effect of diagnosis: all *F* > 4.49, all *p* < 0.04), such that patients had higher levels of TNFR2 transcript and lower levels of IKKβ and NIK transcripts compared to controls (Fig. 4). The increase in TNFR2 mRNA was similar in the high inflammation patient subgroup and low inflammation patient subgroup, while there were significant decreases in IKKβ and NIK mRNA in high inflammation patients relative to low inflammation patients and high inflammation controls (IKKβ: −13%; NIK: −12% relative to high inflammation controls, *p* = 0.04; all other *p* < 0.02) (Fig. 4). The levels of both IKKβ and NIK transcripts, which were specifically lower in high inflammation schizophrenia, were also inversely correlated with IL-1β mRNA and plasma CRP levels in patients (Table 3; Supplementary Fig. 2). In controls, IKKβ mRNA did not correlate with IL-1β mRNA or plasma CRP, but NIK mRNA levels were inversely correlated with CRP in controls (Table 3). After accounting for inflammatory status, there was no longer a main effect of diagnosis for TLR4 or RelB mRNAs (all *F* < 2.90, all *p* > 0.09). However, TLR4 mRNA was still significantly higher in low inflammation patients than in low inflammation controls (*p* = 0.04), though there was no difference in TLR4 mRNA between high inflammation patients and high inflammation controls (*p* = 0.47). Compared to low inflammation controls, high inflammation patients had 15% higher TNFR2 mRNA, 38% higher TLR4 mRNA, 16% lower IKKβ mRNA, 14% lower NIK mRNA, and 40% higher RelB mRNA (all *t* > |3.8 | , all *p* ≤ 0.001).

**Table 3:** Correlations between levels of IKKβ and NIK mRNAs with inflammatory biomarkers in controls and people with schizophrenia.

	Control		Schizophrenia	
	IL-1β mRNA	Plasma CRP (mg/L)	IL-1β mRNA	Plasma CRP (mg/L)
IKKβ mRNA	0.05	−0.18	−0.33**	−0.25*
NIK mRNA	0.04	−0.27*	−0.27*	−0.26*

Data presented are correlation coefficients. Bold values indicate significant correlations. **p < 0.01, *p < 0.05.

We did not detect a diagnostic effect on the expression of IL1R1, TNFR1, CD40, LTβR, TNFR2, IKKα, IκBα, IκBβ, IκBε, HIVEP2, RelA, cRel, NF-κB1, or NF-κB2 mRNAs (all *F* < 2.07, *p* > 0.15), and high inflammation patients did not differ from high inflammation controls in mRNA levels of any of these transcripts (all *p* > 0.08). We found one significant interaction effect between inflammation and diagnosis for LTβR mRNA levels, which were more strongly associated with inflammatory status in controls than in patients (*F*(1,122) = 4.17, *p* = 0.04); however, no significant interaction effects were detected for any of the other NF-κB transcripts measured (all *F* < 3.16, all *p* > 0.08). Correlations between NF-κB pathway mRNAs and cytokine mRNAs in the full cohort can be found in Supplementary Table 4.

### People with schizophrenia and inflammation have worse language abilities than those without inflammation

Within our patient sample, individuals with high inflammation did not differ from low inflammation patients in PANSS positive (*t*(70) = −1.30, *p* = 0.20) or PANSS negative (*U*(70) = 491.00, *p* = 0.21) symptom scores or working memory scores (*t*(70) = −1.19, *p* = 0.24). However, working memory scores were inversely correlated with plasma CRP (*r* = −0.34, *p* < 0.01). Patients with inflammation had worse language scores than those without inflammation (*t*(70) = −1.98, *p* ≤ 0.05), and language scores were inversely correlated with IL-1β mRNA (*r* = −0.28, *p* = 0.01). PANSS negative symptom scores were inversely correlated with IL-1β mRNA (*r* = −0.23, *p* = 0.04).

## Discussion

The findings support the notion that the levels of peripheral NF-κB-related transcripts may be associated with elevations in cytokine mRNAs in schizophrenia. Thus, alterations in the NF-κB activation pathway may be upstream of changes in circulating immune biomarkers such as inflammatory proteins and CRP in (what appears to be) non-infective inflammation in people with schizophrenia. Our results also suggest that levels of only two markers, CRP and IL-1β mRNA, can be used to identify a subset of patients with elevated inflammation and who also have more severe deficits in language abilities. We are the first to report that circulating leukocytes from people with schizophrenia have alterations in the expression of several NF-κB pathway mRNAs that can be grouped into three distinct classes: 1) cell surface receptors, 2) intracellular kinases and 3) regulatory subunits. The changes in cell surface receptor mRNAs are mostly associated with increased peripheral inflammation in both controls and people with schizophrenia. However, one cell surface receptor mRNA, TNFR2, was increased even in people with schizophrenia with ‘normal’ levels of IL-1β mRNA and CRP relative to low inflammation controls. Conversely, the decreased mRNA levels of both the NF-κB kinases, IKKβ and NIK, appear unique to high inflammation patients. We also report that the transcript encoding the regulatory NF-κB subunit RelB is strongly up-regulated by peripheral blood leukocytes in people with peripheral immune activation regardless of diagnosis.

In this study, using a larger sample here, we replicated our previous findings of increased IL-1β mRNA in the blood of people with schizophrenia and lack of difference in IL-6, IL-8 and IL-18 mRNAs between controls and patients in an extended clinical sample (43 controls and 43 patients previously versus 83 controls and 87 patients in the current study) [18]. We should note that we did not find an increase in IL-1β mRNA in one of our earlier studies [5], which may have been due to combined measurements over two different assays which appeared to add increased variability as compared to the single Fluidigm assay used in this study. Here, we additionally report elevated leukocyte expression of IFN-γ—another pro-inflammatory cytokine—at the transcript level in our chronically ill patient sample, which was even more increased relative to healthy controls than IL-1β mRNA. This finding is consistent with IFN-γ protein being a stable ‘trait’ marker of schizophrenia that remains elevated throughout the course of the illness [35], and demonstrates that changes in blood IFN-γ protein are likely a result of increased IFN-γ synthesis by blood leukocytes. This would be consistent with T cell activation and T helper-1-skewed pro-inflammatory immune responses in people with schizophrenia [36] since IFN-γ is generally T cell- or natural killer cell-derived and has pro-inflammatory priming and activating effects on macrophages [37]. However, IFN-γ is also reported to be produced by monocyte-derived macrophages in vitro [38], aligning with findings of elevated macrophage-derived cytokines in patient blood [5, 39–42].

Though there have not been reports of increased IFN-γ mRNA or protein in the brain in schizophrenia, studies have found up-regulation of the viral restriction factor interferon-induced transmembrane protein (IFITM) in the post-mortem brain of patients [3, 43–45]. IFITM is induced by IFN and inhibits viral entry and replication. Although IFITM’s function in the brain in schizophrenia is not understood, elevated brain levels of IFITM has been localized to blood vessels and linked to cortical neuropathology in schizophrenia, including inhibitory interneuron dysfunction [44], pro-inflammatory cytokine expression, blood-brain barrier dysfunction and an increased number of parenchymal macrophages [45]. Together, these findings suggest that infection and/or an IFN-driven immune response in the periphery directly contributes to neuroinflammation and neuropathology in at least some people with schizophrenia. Interestingly, the majority of high inflammation patients in our sample had plasma CRP levels consistent with subclinical inflammation (above the normal cut-off of 2.0 mg/mL [46]), while 10/70 patients had CRP levels consistent with ‘mild’ inflammation (10–50 mg/mL), and no patient had CRP levels that would indicate significant infection or inflammation (>100 mg/mL) [47, 48]. It is therefore plausible that the IFN response in people with schizophrenia reflects mild inflammation driven by a low level of infection, autoimmunity, or tissue injury.

In previous studies, we used peripheral cytokine mRNA levels alone to stratify people into high and low inflammation groups [5, 18]. Here, we took a novel approach and added the commonly used marker (CRP) to our cluster analysis. In doing so, we found that only IL-1β mRNA and CRP were needed to delineate high and low inflammation subgroups, suggesting that these two inflammatory mediators could be the main indicators of peripheral inflammation. Since IFN-γ mRNA was also higher in patients’ blood, we expected that it may help to discriminate the inflammatory subgroups, but it was not selected by the analysis. This may indicate that IFN-γ mRNA is somewhat redundant for immune stratification when also using IL-1β mRNA or CRP. While IL-1β protein can induce CRP synthesis by the liver [49] the main trigger for CRP is considered to be IL-6. We found that IL-6 did not significantly differ between high and low inflammation individuals or between controls and patients in our cohort at the transcriptional level, but IL-6 does typically appear to be elevated at the protein level in people with schizophrenia [5, 16, 50–53]. Interestingly, the stimulatory effect of IL-1β on CRP synthesis by hepatocytes appears to be mediated by an NF-κB-dependent “autocrine IL-6 loop” in the liver [49], meaning IL-6 does not necessarily need to be leukocyte-derived to increase CRP release into the bloodstream and may in fact be liver-derived. Several other studies have, however, found elevated IL-6 mRNA [15–17] in the blood of patients compared to controls, pointing to multiple cellular contributors to increased blood IL-6 levels in schizophrenia.

The findings suggest that, at the transcriptional level, increases in IL-1β persist despite the apparent anti-inflammatory actions of antipsychotics on white blood cells [54, 55], since levels of IL-1β transcript were inversely correlated with antipsychotic dose in patients but still significantly higher in leukocytes from patients than controls. Consistent with this, leukocyte transcript levels of RelA—the subunit that forms part of the most transcriptionally active NF-κB dimer in the human body [56]—were also inversely correlated with antipsychotic dose in our cohort. This implies a putative suppressive effect of antipsychotics on IL-1β expression by peripheral blood leukocytes (likely macrophages) which may be mediated by a reduction in cytoplasmic RelA abundance. However, this putative anti-inflammatory effect of antipsychotics [54] is clearly not sufficient or enduring enough to reduce inflammation in all patients in the long term [55]. Further, our findings suggest that patients off medication would be expected to have even higher levels of IL-1β than those who are medicated and clinically stable. The strength of the correlations between CPZ and RelA mRNA, and CPZ and IL-1β mRNA reported here are weak [57], and the finding that high and low inflammation patients did not differ significantly on daily antipsychotic dose supports a negligible effect—if any effect—of antipsychotic medication on inflammation in our cohort.

We note that diagnostic differences reported here in TLR4 mRNA, TNFR2 mRNA and NIK mRNA levels were highly significant with medium effect sizes prior to multiple comparisons correction and are therefore unlikely to reflect type 1 errors. Further, altered expression and function of TLR4 and members of the TNF family in blood have been implicated in schizophrenia previously (discussed below), supporting the likelihood that our current findings in TLR4 mRNA, TNFR2 mRNA and NIK mRNA levels are not spurious but biologically meaningful. That people with schizophrenia have increased leukocyte transcription of the receptor TLR4 that activates NF-κB is supported by the current findings, and even patients with low inflammation had significantly higher TLR4 mRNA than controls with low inflammation. This might suggest that some degree of increased TLR4 expression by leukocytes is inherent to schizophrenia. Since TLR4 is an innate immune receptor for bacterial glycoproteins and a major activator of the M1 macrophage phenotype [58], our findings are consistent with high levels of macrophage-derived cytokines in patient blood [5, 39–42], and with other studies finding increased TLR4 mRNA in peripheral mononuclear cells extracted from people with schizophrenia [19, 20]. Together with previous findings that TLR4 activation in patient immune cells leads to exaggerated pro-inflammatory cytokine release [21], higher TLR4 mRNA in white blood cells would be consistent with a higher number of TLR4 receptors at the cell surface in schizophrenia. Intriguingly, an increase in IFN-γ—as we report here—primes macrophages for stimulus-induced secretion of pro-inflammatory cytokines via enhancement of TLR4 signaling [59, 60], further supporting that people with schizophrenia are positioned to over-respond via TLR4. Overall, TLR4 up-regulation in patient circulating leukocytes plausibly contributes to increased peripheral inflammation in a subset of people with schizophrenia. However, this raises the interesting question of what else may be inducing increased innate immune receptor TLR4 mRNA in white blood cells in schizophrenia, with the possibility that there may be a higher bacterial load [61, 62].

The other NF-κB-activating receptor mRNA that was increased in patients encodes TNFR2, a non-canonical receptor that is uniquely expressed on immune cells [63] and can only be fully activated by transmembrane, not soluble, TNF [64]. TNFR2 transcript levels were higher in both high and low inflammation patients compared to both high and low inflammation controls. This suggests that changes in leukocyte expression of TNFR2 mRNA are more related to a diagnosis of schizophrenia than to inflammatory status. TNFR2 competes with TNFR1 for the cytoplasmic pool of intermediary proteins linking TNFR stimulation to NF-κB activation [63]. However, unlike TNFR1, TNFR2 has no intrinsic cell death-inducing activity [65]. Our finding of increased TNFR2 mRNA in the blood of patients therefore suggests that TNF signaling in people with schizophrenia is slanted toward pro-survival immune cell activation that may promote immune cell survival and/or sustain inflammation. However, while TNFR2 is considered as a cell membrane protein, the extracellular portion can also be cleaved and form soluble TNFR2 (sTNFR2) - with increases in sTNFR2 in patient plasma reported [66, 67] - and sTNFR2 can bind to soluble TNF in the blood to either amplify or neutralize its activity [68]. Elevated circulating (soluble) TNF protein is consistently found in chronically-ill people with schizophrenia across multiple cohorts [5, 15, 35, 69]. Our finding of up-regulated TNFR2 mRNA in the circulating leukocytes of patients may therefore contribute to both pro- and anti-inflammatory processes: if the mRNA findings translated into higher levels of membrane-bound TNFR2 receptors on leukocytes (pro-inflammatory) or if they translated into higher levels of circulating sTNFR2 (pro- or anti-inflammatory). While we did not find evidence to suggest higher mRNA levels of the TNFR2 ligand TNF-α in schizophrenia, increased TNF-α protein in the blood of patients has been reported previously, including in the current clinical cohort [5, 70, 71]. Thus, the TNF protein elevations found in circulation may derive from cells other than white blood cells.

Given that NF-κB kinases are necessary for forward propagation of the NF-κB signal from receptor to nucleus [72], it may appear counterintuitive that a subset of people with schizophrenia had downregulated peripheral expression of IKKβ and NIK transcripts but increased expression of IL-1β mRNA and CRP. This is in contrast to the brain, where we found the expected strong, positive relationship between NF-κB kinase mRNAs and proinflammatory cytokine mRNAs overall [14]. Our results here suggest that synthesizing less IKKβ and NIK mRNA may potentiate, not dampen, inflammation in the periphery in people with schizophrenia. In support of this possibility, anti-inflammatory functions of NF-κB kinases in immune cells have been reported [73–75]. In mice, IKKβ-deficient macrophages and neutrophils secrete significantly more IL-1β upon immune challenge than wild-type cells [76]. This is particularly interesting given that patients in our cohort had higher levels of IL-1β transcript in the blood, and several studies have found elevated IL-1β protein in the blood in people with schizophrenia compared to controls [15, 41, 53, 77]. Macrophages lacking IKKβ may therefore maintain a pro-inflammatory M1-like phenotype with sustained cytokine release once inside tissue and fail to switch to an M2 anti-inflammatory state [73, 74]. NIK, like IKKβ, can also negatively regulate the expression of cytokines in immune cells, since NIK knockout mice have an exaggerated IFN response to infection [75]. This is pertinent given our findings of decreased NIK mRNA and increased IFN-γ mRNA in people with schizophrenia relative to controls, and suggests that NIK deficiency could also sustain the M1 macrophage phenotype.

In terms of genes encoding NF-κB subunits, we found that peripheral blood leukocyte levels of RelB transcript in patients were higher than controls. However, the strength of this effect was more marginal compared to diagnostic differences in other NF-kB pathway mRNAs such as TLR4, TNFR2, IKKβ and NIK and did not survive multiple comparisons correction. It is possible that this change in RelB mRNA in the blood of people with schizophrenia compared to controls reflects type 1 error. Additionally, further stratifying our cohort based on inflammatory status revealed that this elevation not only occurs in patients with inflammation but also occurs to the same degree in controls with inflammation. As such, it appears that RelB could be a more general marker of elevated inflammation and NF-κB activation.

An important consideration in the interpretation of our findings is the magnitude of transcriptional changes in peripheral leukocytes of people with schizophrenia. It has been reported that a fold-change above 1 is required for a specific transcriptional change to be considered ‘biologically meaningful’ [78–80], while the current findings range from 7% for TNFR2 mRNA to 42% IFN-γ mRNA. Though this may suggest that IL-1β and IFN-γ mRNAs increases in schizophrenia are mild to moderate, we would argue that large changes would not necessarily be expected in vivo in medicated patients with schizophrenia who appear to have low-level, subclinical inflammation. However, they still could be ‘biologically or clinically meaningful’. Indeed, elevated cytokine transcription and plasma CRP in our patient cohort is associated with worse psychiatric and cognitive symptoms [18, 25], strengthening the contention that even minor changes in cytokine transcription clinically relevant in schizophrenia. Regarding NF-κB transcripts, NF-κB activation is tightly regulated to prevent life-threateningly excessive immune activation and our gene expression results suggest that many transcripts in the NF-κB pathway are expressed in a narrower range than many of the cytokines. Thus, changes of very large magnitude in NF-κB transcripts may not occur in vivo, or may only occur under conditions of severe infection and/or inflammation. Given that NIK mRNA was positively correlated with plasma CRP in both control subjects and people with schizophrenia, and IKKβ mRNA was also correlated with CRP in patients, it is entirely plausible that small changes to the steady-state levels of these transcripts have amplified downstream effects on immune signaling. Finally, it is also possible that the changes reported here are not occurring universally across all leukocytes but rather in a specific immune subset such as macrophages. Putative cell-specific changes may be dampened in assays containing all peripheral blood leukocytes, and single-cell RNA studies would be needed to address this.

Although we were unable to covary for BMI in our analyses given that we had BMI data for only a small number of controls, we were able to assess the relationship between BMI and inflammation markers in the patients, a group in which we would be more likely to find such a relationship since healthy controls would be less likely to have either inflammation or obesity [81]. However, the findings do not support a strong role for BMI in the differences observed in inflammatory markers in high versus low inflammation within the patient subgroups. BMI did not differ between high and low inflammation groups, and only 3 inflammatory markers were weakly associated with BMI across the entire cohort of schizophrenia patients. CRP was positively, but weakly, correlated with BMI in patients, consistent with various studies showing CRP is increased in overweight and obese individuals [82]. Apart from IFN-γ, which was negatively correlated with BMI, there was no association between BMI and any other pro-inflammatory cytokine measured, including IL-1β, which was a major determinant of inflammatory categorization in our cohort. Therefore, increased BMI does not seem to be the sole explanation for our findings. Future studies should confirm the contention that BMI does not overtly influence NF-κB pathway changes in patients with schizophrenia.

Taken together, the findings confirm that people with schizophrenia have altered expression of transcripts involved in NF-κB activation in blood, which may relate to chronic inflammation that drives PFC-dependent cognitive symptoms in a subset of patients. Moreover, we have shown that stratifying patients based on levels of IL-1β mRNA and CRP in the blood reveals some aspects of NF-κB dysregulation that are common to all people with schizophrenia and others that are unique to people with schizophrenia with inflammation. Thus, stratifying based on inflammatory status is potentially useful in understanding the mechanisms behind peripheral inflammation in this patient subgroup and may help identify patients who would most benefit from adjunctive anti-inflammatory medication. It appears that people with schizophrenia are primed for exaggerated and prolonged immune responses via higher transcription of NF-κB-activating receptors at the level of the cell membrane, and that some patients are potentially are less able to attenuate NF-κB signaling due to lowered synthesis of key intracellular regulators, IKKβ and NIK. Overall, our findings support the prime importance of the pro-inflammatory cytokine IL-1β and of CRP in inflammation-associated schizophrenia and implicate altered expression of regulatory NF-κB pathway mRNAs in driving peripheral inflammation, and possibly cognitive impairment, in a substantial proportion of patients with schizophrenia.

## Supplementary information

Supplementary material
