# Supplementary material for: Peripheral NF-κB dysregulation in people with schizophrenia drives inflammation: putative anti-inflammatory functions of NF-κB kinases
Source: Transl Psychiatry. 2022 Jan 13;12:21. doi: 10.1038/s41398-021-01764-2 (PMC8758779; doi:10.1038/s41398-021-01764-2)
Supplement: Supplementary file 1 — Supplementary material [file 41398_2021_1764_MOESM1_ESM.docx]

**Supplementary material**

*Supplementary Table 1***.** Relationships of NF-κB transcripts in blood with demographic variables

|  | Control  (N=70-77) | | Schizophrenia  (N=77-82) | |
| --- | --- | --- | --- | --- |
| *mRNA* | *Age* | *RIN* | *Age* | *RIN* |
| *IL-6* | 0.22 | 0.14 | 0.01 | -0.12 |
| *IL-1β* | 0.17 | 0.22 | 0.08 | -0.21 |
| *IL-8* | -0.13 | -0.08 | 0.17 | 0.10 |
| *IL-18* | -0.07 | 0.04 | **0.23*** | 0.07 |
| *TNF-α* | 0.21 | -0.06 | 0.18 | **-0.34**** |
| *IFN-γ* | 0.05 | **-0.34**** | -0.11 | 0.11 |
| *IL1R1* | 0.05 | **0.32**** | -0.10 | **0.45***** |
| *TNFR* | -0.02 | -0.02 | 0.05 | -0.09 |
| *TLR4* | -0.11 | 0.21 | -0.04 | **0.29**** |
| *CD40* | -0.02 | 0.05 | -0.05 | -0.16 |
| *LTβR* | 0.13 | -0.09 | 0.17 | **-0.27*** |
| *TNFR2* | 0.20 | 0.07 | 0.22 | **-0.27*** |
| *IKKα* | -0.10 | **0.39**** | -0.14 | **0.32**** |
| *IKK**β* | 0.12 | 0.10 | **-0.25*** | 0.14 |
| *NIK* | 0.11 | 0.07 | -0.03 | -0.21 |
| *IκBα* | -0.20 | **-0.37**** | 0.14 | **-0.49***** |
| *IκBβ* | -0.22 | <0.01 | 0.19 | **-0.52***** |
| *IκBε* | -0.02 | **-0.24*** | 0.08 | **-0.32**** |
| *HIVEP2* | 0.02 | 0.10 | -0.16 | -0.16 |
| *NFKB1* | <0.01 | -0.03 | 0.06 | **-0.57***** |
| *NFKB2* | 0.07 | **0.32**** | -0.19 | 0.06 |
| *RelA* | 0.06 | -0.11 | 0.13 | **-0.38**** |
| *RelB* | -0.03 | -0.01 | 0.05 | -0.21 |
| *cRel* | 0.07 | 0.22 | -0.04 | **0.32**** |

RIN, RNA integrity number. Data presented are correlation coefficients. Bold values indicate significant correlations. ***p<0.001, **p<0.01, *p<0.05.

*Supplementary Table 2***.** Relationships of BMI and inflammatory variables in patients

|  | Schizophrenia  (N=55-59) |
| --- | --- |
| *Inflammatory variable* | *BMI* |
| *CRP protein* | **0.29*** |
| *IL-6 mRNA* | 0.14 |
| *IL-1β mRNA* | 0.16 |
| *IL-8 mRNA* | <0.01 |
| *IL-18 mRNA* | 0.07 |
| *TNF-α mRNA* | 0.22 |
| *IFN-γ mRNA* | **-0.29*** |
| *IL1R1 mRNA* | -0.13 |
| *TNFR mRNA* | 0.03 |
| *TLR4 mRNA* | -0.10 |
| *CD40 mRNA* | -0.06 |
| *LTβR mRNA* | 0.10 |
| *TNFR2 mRNA* | -0.06 |
| *IKKα mRNA* | -0.13 |
| *IKKβ mRNA* | -0.22 |
| *NIK mRNA* | <0.01 |
| *IκBα mRNA* | 0.16 |
| *IκBβ mRNA* | 0.23 |
| *IκBε mRNA* | **0.27*** |
| *HIVEP2 mRNA* | 0.02 |
| *NFKB1 mRNA* | 0.19 |
| *NFKB2 mRNA* | -0.04 |
| *RelA mRNA* | 0.10 |
| *RelB mRNA* | **0.28*** |
| *cRel mRNA* | 0.25 |

BMI, Body Mass Index. Data presented are correlation coefficients. Bold values indicate significant correlations. *p<0.05.

*Supplementary Table 3.* Comparison of demographic and clinical variables between high and low inflammation patients

|  | **Low inflammation SCZ**  (n=46) | **High inflammation SCZ** (n=26) |  |  |
| --- | --- | --- | --- | --- |
|  |  |  | t/U/X^2^ (df) | p-value |
| Age (years) | 36.3 (8.6) | 35.0 (8.4) | t(70)= -0.63 | 0.54 |
| Age of onset (years) | 22.5 (5.4) | 23.4 (7.0) | U=584.50 | 0.87 |
| Duration of illness (years) | 14.0 (8.1) | 11.4 (7.0) | U=491.50 | 0.21 |
| BMI | n=37  32.4 (6.7) | n=21  29.9 (6.1) | t(56)=1.42 | 0.16 |
| Male/female | 33/13 | 10/16 | χ^2^(1)=7.65 | **0.006** |
| CPZ equivalent (mg)/day | 560.3 (483.0) | 563.7 (495.1) | U=588.00 | 0.91 |
| Clozapine/other | 15/31 | 11/15 | X^2^(1)=0.68 | 0.41 |
| Typical/atypical | 3/43 | 2/24 | X^2^(1)=0.04 | 0.85 |

SCZ, schizophrenia; BMI, body mass index; CPZ, chlorpromazine. Data presented as mean (standard deviation). Bold values indicate significance at p<0.01.

*Supplementary Table 4*. Relationships of leukocyte NF-κB pathway mRNAs with leukocyte cytokine mRNAs in whole cohort

|  | *IL-6* | *IL-8* | *IFN-γ* | *TNF-α* | *IL-1β* | *IL-18* |
| --- | --- | --- | --- | --- | --- | --- |
| *IL1R1* | -0.18 | **0.25**** | **0.28**** | **-0.35***** | 0.10 | 0.04 |
| *TNFR* | -0.01 | 0.15 | 0.16 | -0.13 | **0.44***** | 0.04 |
| *TLR4* | **-0.19*** | **0.28**** | **0.23*** | **-0.23*** | **0.34***** | 0.10 |
| *CD40* | **0.49***** | -0.16 | -0.07 | 0.06 | -0.02 | <0.01 |
| *LTβR* | 0.10 | -0.09 | **0.20*** | 0.08 | **0.43***** | **0.27**** |
| *TNFR2* | 0.01 | **0.19*** | 0.06 | 0.04 | **0.47***** | **0.24**** |
| *IKKα* | 0.05 | 0.01 | -0.14 | -0.10 | <0.01 | 0.01 |
| *IKKβ* | 0.13 | -0.14 | **-0.19*** | 0.06 | **-0.24**** | 0.07 |
| *NIK* | **0.28**** | 0.04 | -0.10 | 0.06 | **-0.20*** | 0.13 |
| *IκBα* | 0.09 | 0.03 | **0.18*** | 0.14 | **0.41***** | 0.12 |
| *IκBβ* | **0.23*** | -0.08 | -0.11 | **0.26**** | **0.33***** | 0.08 |
| *IκBε* | **0.21*** | -0.12 | -0.11 | **0.40***** | **0.18*** | **0.20*** |
| *HIVEP2* | 0.07 | -0.02 | -0.13 | 0.02 | 0.02 | -0.13 |
| *NFKB1* | **0.24**** | **-0.21*** | 0.09 | 0.12 | **0.48***** | 0.03 |
| *NFKB2* | 0.04 | 0.11 | -0.11 | -0.01 | **0.35***** | 0.06 |
| *RelA* | 0.07 | 0.07 | -0.04 | 0.13 | -0.07 | 0.05 |
| *RelB* | 0.22** | -0.03 | 0.08 | 0.09 | **0.48***** | 0.18 |
| *cRel* | 0.12 | 0.13 | -0.13 | -0.08 | -0.17 | **0.31***** |

Data presented are correlation coefficients. Bold values indicate significant correlations. *p<0.05, **p<0.01, ***p<0.001.

*Supplementary Figure 1*. Relationship of IL1β mRNA with antipsychotic dose in patients. IL1β mRNA is presented as non-transformed data though was log-transformed for analyses.


*Supplementary Figure 2*. Relationships of NIK and IKKβ mRNAs with IL1β mRNA and CRP protein in patients. IL1β mRNA is presented as non-transformed data though was log-transformed for analyses.
